# Providing ‘professionalism with compassion’; how the time for caring communication can improve experiences at the end-of-life at home, findings from a realist evaluation

**DOI:** 10.1186/s12904-024-01610-4

**Published:** 2024-12-21

**Authors:** Kathryn McEwan, Joanne Atkinson, Amanda Clarke, Angela Bate, Caroline Jeffery, Sonia Dalkin

**Affiliations:** University of Northumbria at Newcastle, Newcastle Upon Tyne, England

**Keywords:** Palliative care, End-of-life care, Public health, Communications, Rapid response services, Realist evaluation, Transitions theory

## Abstract

**Background:**

For many patients and caregivers, attending to dying and death at home will be a new and fearful experience. This research brings new evidence on the central support of the Rapid Response Service (RRS), provided to those who chose to die at home. RRS’s are variable, although all seek to avoid unwanted hospital admissions and to respond flexibly to suit individual preferences for support. Staffed by specialist palliative and end-of-life care nurses, the RRS works alongside primary and acute care, but little is known on their impact.

**Methods:**

Realist evaluation is a theory driven approach which identifies patterns of generative causation; this approach ascertains what works, for who, why, and in what circumstances. In this study, initial theories were developed by the research team and subsequently tested through semi-structured realist interviews with patients, caregivers, RRS staff, and other health practitioners. Iterative rounds of data analysis were undertaken to tease out contexts, mechanisms and outcomes, testing and revising the theories, including the application of substantive theory. Finally, we produced refined programme theories (PTs) which provide the basis for wider application of findings.

**Results:**

Overall, 36 participants contributed, and six areas of inquiry were developed. This paper sets out the data from one area, PT Skilled Communication. Several benefits arose from RRS staff having the time to talk to and with patients and caregivers: specifically, that this communication improved knowledge in a tailored and compassionate manner. These exchanges allayed fears and reduced uncertainty, improving confidence to care. This was particularly embedded in one RRS because of their holistic approach.

**Conclusions:**

Supporting death and dying at home is a novel and difficult experience for many; skilled communication, provided by specialist palliative care staff, can make a positive difference. Through their provision of compassionate support, RRS staff can help caregivers to recognise and respond to different symptoms and situations, reducing fear. By responding rapidly, only on request, they also meet individual preferences for contact. Together, this improves the opportunity for the last days and hours of life to be experienced, at home, in as affirmative a manner as possible.

**Supplementary Information:**

The online version contains supplementary material available at 10.1186/s12904-024-01610-4.

## Background

When people choose to die at home, they are often supported by community end-of-life care services. These services aim to enable patient choice to maximise their days at home for as long as it feels right, to work collaboratively across primary care to reduce service delivery pressures, and to cut the risk of unplanned hospital admissions [1–6]. Services such as these are variable across the UK, with diverse service models operating across different regions; one form of community end-of-life care service is the Rapid Response Service (RRS). These services are similarly variable and operate as outreach from acute care or are commissioned from the third sector although both service models work alongside general practitioners (GPs) and district nurses (DNs). RRS’s are flexible and responsive services, offering to attend the home as often, or as little, as any individual’s preference. For those who wish to die alone, or who wish to retain their privacy and time together with chosen friends and family [7], the RRS’s flexibility allows for fewer and shorter responses, so providing an efficient and authentically person-centered response.

A recent Marie Curie report [8], found that community palliative and end-of-life care is variable across the UK. As a result, too many people are not receiving the care and support they need when they need it [8]. Further, that informal caregivers are often under great strain while supporting end-of-life, causing short-term distress and long-term trauma [8]. Differing RRS offer variable packages of care and access. In addition, they diverge in the time made available to communicate with patients and caregivers, as well as their approaches to service access and engagement, all of which may contribute to fluctuating potential for supporting death preparedness. For many patients and caregivers who access the RRS, witnessing death and dying will be a new experience on which they have few resources to draw [9]. Our understandings of death and dying are heavily influenced by socio-cultural values, political beliefs, and historical experiences [10, 11]. Historically, death and dying took place in the home [10] yet more recently, the sequestration [12] and medicalisation of death has moved death and dying into hospitals, meaning many people have lost the opportunity to build skills and experience of understanding and responding to ordinary dying [13]. While such segregation continues, it is recognised that early comprehensive communication strategies that include a ‘triad of experts’, such as patient, caregiver, and specialists, can improve outcomes [14]. Honest and timely communications around death and dying are important to patients and their caregivers [15] and, as such, this is recognised in the National Institute for Clinical Excellence (NICE) guideline: Care of dying adults in the last days of life (NG31) [16].

When caregivers are unprepared for death, they suffer more depression, anxiety, and complicated grief [17]. Caregivers often experience uncertainty when supporting at the end-of-life, yet communication, that includes clear and reliable information, can reduce their hesitancy [18]. A recent report from NCEPOD identified the availability of staff to provide individualised communication, so delivering person centred care, as good practice and an area for improvement in palliative and end-of-life care services [19]. A trusted relationship between caregivers and healthcare professionals, allows for the individual tailoring of communication to meet needs, establishing genuine understanding, and providing useful tools for action [18]. Time is an important resource in end-of-life care [20] and is required for personalised compassionate communication on the realities and practicalities of supporting death and dying at home, and to ensure understanding [21]. Dying people may fear being helpless, dependant, and in pain, clear conversations with practical advice are suggested to manage these fears [22, 23].

In a hospital setting, effective communication is one of the top five elements identified by patients and their families as important to their care, alongside respectful and compassionate care [24]. In this study, we explore this in a community setting, seeking new evidence on the form and impact of the skilled communication provided by the RRS staff, in a compassionate manner, to patients and caregivers at the end-of-life at home. In doing so, we incorporate transitions theory [25], recognising that death, dying, and bereavement are significant life transitions. We posit that the lack of death literacy in our society [9], has reduced our common knowledge of what to expect and how to navigate these final life transitions [13]. As such, expectations of the expert patient, or in this case perhaps expert informal caregiver [26], are particularly excluding at home at the end-of-life.

In this article, we set out findings from a realist evaluation on the form and practise of skilled communication from specialist staff at the end-of-life at home, and the perceived impact of this from the perspective of patients, caregivers, and RRS staff.

## Methods

### Realist evaluation

Realist evaluation is a theory driven approach used to understand complex programmes (i.e., interventions, services, policies); it accounts for key contexts within which—and mechanisms through which—outcomes are achieved [27]. Applying realist approaches, offers the potential to describe a pattern of outcomes for RRS’s for end-of-life care, in complex social systems [28] through focusing on ‘what works, for who, in which circumstances’. Realist evaluation attends to the ways that programmes may have different effects for different people, depending on the contexts into which they are introduced. An intervention or service for people with end-of-life care needs, is considered to provide resources that alters the context into which it is introduced [29] triggering a change in the reasoning of patients and their caregivers; this leads to a particular outcome, i.e. Context + Mechanism = Outcomes (or CMOs). CMOs are used as explanatory formulae (otherwise referred to as realist programme theories), which are developed and used as a ‘lens’ to focus data collection and interrogate the data. They are refined as the project progresses and once refined, in effect, postulate potential causal pathways between programmes and impacts.

### Study design

The study incorporated three phases. Phase one was the ‘theory gleaning’ phase [30] wherein we developed initial programme theories (IPT) of what, why, and how the RRS may work through research team meetings, Patient and Public Involvement (PPI), stakeholder discussions, and literature scoping (for a full exposition of the approach taken and the results of that specifically for this phase, see our earlier methods paper [31]). Phases two and three were undertaken concurrently; these included focus groups and semi-structured interviews with practitioners, patients, and caregivers on two identified sites to collect qualitative data, alongside the development and application of a health service use log and the analysis of routine service use data.

Ethics were obtained by the Health Research Authority (HRA) through the Research Ethics Committee (REC) (Haydock Ref: 21/NW/0256). Once approved, ethics were then agreed with the Research and Development Office of the Trust in Model A and the Charity Head Office in Model B.

### Data collection

Informed consent was gained from each participant in advance through sharing of participant information sheets and consent forms, as all interviews and focus groups were conducted over Microsoft Teams and Telephone. Where forms could not be easily signed and returned, we took verbal consent, confirming the forms had been read and understood at the beginning of the interview and through email or telephone contacts to set up the date and time.

The interviews followed a series of topics and statements drawn from the IPTs to begin to test these suppositions, in line with the realist interview approach [32]. Qualitative data was transcribed verbatim and added to NVivo 12 Pro [33] for the analysis process to identify various contexts, mechanisms, and outcome configurations in the data, using a realist logic of analysis [34]. The wider research team and PPI representatives, interrogated the identified patterns identified throughout the research process, addressing contradictions and correlations, allowing the refinement of the programme theories. The study results are reported in line with RAMESES reporting guidelines for realist evaluation [35] see Supplementary File 1 for checklist.

### Sample

Two sites were included in data collection (see Table 1 for an overview of site characteristics), these were identified in advance of the project commencement through principal investigators (PI) networks and contacts.

**Table 1:** Site characteristics for model A and model B

Characteristics	Model A- RRS	Model B- RRS
First Port of Call Protocol	Patients/Caregivers are to contact DN’s in first instance as service gatekeeper Should they not answer/be able to respond and/or is crisis are able to directly contact RRS	Patients/Caregivers are to contact DN’s in first instance as service gatekeeper Should they not answer/be able to respond and/or is crisis are able to directly contact RRS
Days/Hours of Service	12 h a day/7 days a week	24 h a day/7 days a week
Situation	Out of hours service adjunct to planned day service	In and out of hours holistic service
Serves	Patients already in acute palliative service. Patients on palliative register in one locale	Patients and caregivers in need at the end-of-life in one GP locale
Staffed	One specialist palliative care nurse from planned service covers on rota	One specialist palliative care nurse and one senior health care assistant always available
Referrals	From planned service, from GP or DN, self-referral feasible but not advertised	From GP or DN, self-referral is feasible, and leaflets left in local surgeries

All staff on both sites were invited to participate in interviews, this was not limited by our sampling, we interviewed and included all who were interested and were able to find the time to engage. We used snowball sampling from RRS staff and wider networks in palliative and end-of-life care in the region to invite other health and social care practitioners. These were our external staff who worked alongside the RRS and could bring a wider perspective. Patients and caregivers were first approached by RRS staff as research gatekeepers; they introduced the study and asked for permission to share contact details with the researcher (KM). The research team then attempted to recruit directly from these shared contacts.

Overall, 36 participants contributed to the qualitative data (see Table 2). Only one staff focus group occurred on one site (Model B); following this, all the subsequent planned focus groups were converted to single interview contacts, since this was more convenient for staff.

**Table 2:** Qualitative data breakdown

	Permission to Contact Details Referred	Interviews completed
**RRS Staff (FG + Interview)**	**Caregivers**	**Caregivers**
Service Model A **6**	Service Model A **8**	Service Model A **1**
Service Model B **14**	Service Model B **13**	Service Model B **5**
**External Staff (Interview)**	**Patients**	**Patients**
Service Model A **6**	Service Model A **5**	Service Model A **0**
Service Model B **3**	Service Model B **2**	Service Model B **1**

### Data analysis

Following the realist evaluative approach set out above, we followed an iterative approach to analysis [28, 30]. As interviews were undertaken, excerpts from transcripts were shared with the wider research group and discussed in meetings which included our PPI representatives. Contexts, mechanisms, and outcomes were teased out in these in-person and Microsoft Teams meetings, and programme theories were revised concurrently. See Supplementary File 2 for a full development of IPT, through various identified CMO configurations, revised PTs, and final refined PT.

**Fig. 1:**
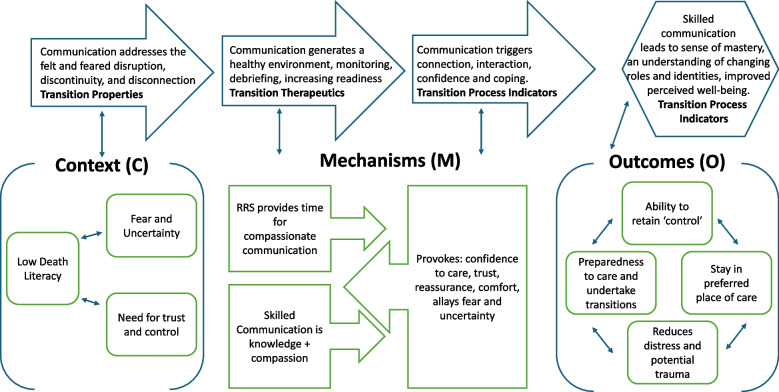
Skilled Communication—CMMO & Transitions Theory

A retroductive approach to analysis was undertaken to dig deep into the data over multiple rounds of analysis to understand the causal relationships [31, 36]. As part of this iterative retroductive investigation, substantive theories were considered for explanatory value. Transitions theory [25] was the substantive theory which guided the theorising throughout. This theory was selected as it recognised the variety of resources which need to be available for people to manage significant change and make successful transitions into new ways of being. We worked the various aspects of transitions theory into a Microsoft Excel spreadsheet with questions that prompted us to consider through the iterative rounds of analysis whether we were including these frames of reference. The influence of this on the results is identified in the results section and summarised in Fig. 1, it is also identified in the discussion section, to view how it also underpinned the analysis, see Supplementary File 5.

## Results

In the following section, we set out the data that underpins the final refined programme theory (PT) (Table 3) and our subsequent recommendations (Table 4) for this one investigative area of a larger study. The final refined programme theory and the embedded substantive theory, transitions theory, is set out visually in Fig. 1.

The different components of the context, triggering mechanism (resource and response), and subsequent outcome in the final refined programme theory are outlined with relevant evidence from primary data collection. Whilst this is provided in discrete sub-sections (e.g., ‘Context’), it is noted that there is overlap between components, highlighting generative causation throughout. This is shown through labelling of context, mechanism, and outcome throughout the participant quotes.

The influence of our substantive theory, transitions theory [25], is described at the end of this section and summarised visually in Fig. 1.

### Low death literacy and fear (context)

Our death literacy is measured through our familiarity with concepts and practicalities of death and dying; it includes how well we understand the processes involved, and so, resultingly how equipped we are to make choices and decisions. Predominantly, death literacy is low in the United Kingdom (UK), as such, in the interviews, RRS staff and caregivers explained to us concerns, or fears, that arose from the unknowns of care at the end-of-life. This uncertainty impacted on caregivers interactions with the RRS, their decision making, and their experiences of supporting death and dying at home. For example, fearing that medication might be purposefully given to hasten death.

Rapid Staff Model B: *[a caregiver said] “you’re not going to give her anything that’s gonna make her die”* (context) (RS8)

RRS staff provided examples of how low death literacy, fear, and lack of trust can all contribute to patients and caregivers worries about allowing the RRS into their home. They feared this could result in them ‘losing control’ of supporting death and dying at home.

Rapid Staff Model B**:*** a family recently […] heard about the syringe driver [and] said we're not having that because then they'll die. […].* (context) *[…] they feel that they don’t like to hand over control and we definitely don't want to take over their control anyway* (context)**.** (RS5)

This RRS staff member went on to identify how they could use their available time and communication skills to respond to difficult situations and build trust.

Rapid Staff Model B**:** *So, there is all of that built up frustration and anger and mistrust* (context) *[and] you come to crisis point, [when families] feel that they're dealing with it now, themselves, because they're in their own home. […]. We spend the time* (resource) *[whereas] they can sense that the district nurses and the GPs rush* (response) *[but] we are here for as long as you need us to be* (resource). (RS5)

Caregivers indicated that death and dying was frightening and concerning. They explained how the RRS staff could communicate practically and compassionately improving their understanding and ability to manage care, improving their death literacy.

Caregiver Model B: *[It was] very distressful for eight days to hear the noises she was making* (context). *[…] [T]he nurses assured us that's just a process that 90% people go through* (response) *[the nurses offered] kindness and understanding the situation and [they] know exactly what's going on. You know, knowing what's natural* (resource), *I didn't have that knowledge* (context) *[…] they had that knowledge to sort that out, you know* (resource). (FFC19)

### Availability to provide ‘skilled communication’ (mechanism- resource)

We identified two mechanism- resources regarding time. Firstly, the impact of the availability of RRS staff to patients and caregivers. Secondly, that this time availability allows for the utilisation of skilled communication, which includes knowledge and compassion.

#### Availability/time

When RRS staff attend home visits, they are not restricted by time limits for visits, as other services (i.e., GPs or DNs). RRS staff explained how, unlike the DN model, they do not operate on a caseload basis, with significant volumes of calls to make on that caseload each shift, with time limits imposed on visits as a result.

Rapid Staff Model B: *the best part is that we have time, […] time with the patients and their families […]. [W]e take one call and stay with that call as long as we are needed, […] we don’t back calls up* (resource). (RS10)

Although both Model A and Model B have similar flexibility in terms of time on visits, the Model B service provided an additional role, to attend for verification of expected death (VOED) which Model A did not. As Model B staff respond to VOED they were not under the time pressure that DN/GP would have for this task; this meant they could continue to share compassionate communication during this difficult time of very early bereavement.

Rapid Staff Model B: *[I say] I’m going to be here at least 45 minutes. If there is anything you need to ask while we are here, don’t feel like we are rushing away, you’ve got plenty of opportunity* (resource)*.* (RS10)

RRS staff described how the time their service model provided, allowed them to build relationships through repeated visits. One Model B RRS staff member explained this consistency allowed them to “*build rapport*” (resource) and offered opportunity to get to “*build a familiar feeling*” (resource). This provided space for levity and psycho-social support; “*we’re not all doom and gloom and we chat, and we might have a bit of a laugh*” (response) (RS6).

Caregivers told us that taking away time pressure on visits was valuable; one struggled to put the impact into words and instead physically gestured by demonstrating a sigh of relief. The time availability and subsequent rapport allowed for confidence and reassurance building through skilled communication; RRS staff could increase knowledge through *“discussions on what to expect”* (resource), they could *“take the time to work out what we were feeling”* (resource) and encourage through providing “*praise for what we were doing”* (resource) (FFC6).

Caregiver Model B:* just somebody taking the time to explain to us who they were, what they were about* (resource) *[that] made […] looking after mum, so much easier […]* (outcome).* [RRS offered] professionalism with […] compassion […] and time, they’ve got the time to do it […], or it seems like they have the time to give it to you* (resource) (FFC6).

As time becomes increasingly pressed, for example where a patient is deteriorating quickly at home, RRS staff explained that they could take telephone calls directly to avoid overly complex routes to care. This reduced fear and potential panic from caregivers and avoided emergency calls and unnecessary hospital admissions.

Rapid Staff Model B:* if we're aware that somebody could deteriorate quickly* (context)*, we do say to them look, don’t ring 999* (outcome)*, just ring the nurses, the district nurses or our team, one of us will come* (resource)*. Don’t panic, because they do automatically panic* (response),* but you know, if the DNR has been discussed properly and they've got an emergency healthcare plan* (resource),* then they do tend to understand there's no point ringing 999* (response). (RS5)

The lack of time restraints on RRS teams meant that skilled communication could be enacted.

#### Skilled communication

As Model A is an adjunct RRS, running alongside a planned palliative service, RRS staff also have contact with patients with palliative care needs. For RRS staff in Model B, as their service is only available to patients in the last days of life, they have slightly different considerations of who should communicate what, and when.

Rapid Staff Model A: *[in planned service is a] chap who’s quite newly diagnosed [with] pancreatic cancer, [so feels she] can’t go wading in and talking about what death looks like* (resource) (RS1).

One caregiver, who was very recently introduced to the Model B service at the time of study participation described being somewhat gently introduced to discussions of death and dying by RRS staff who had visited to date. This participant explained they had discussed ‘*treatment*’ rather than ‘*advice about the future and […] what might happen and when*.’ (resource) (FFC1). This tentative approach perhaps reflected his unspoken desire to pace information, as he shared, he was not sure he was prepared to hear more than that at his current stage.

The data suggested that RRS in Model B were able to assess individual situations and respond appropriately, tailoring the amount and type of information shared at different times. For example, whereas the previous caregiver had not wanted to know much yet, and staff had responded to that identified need; another, explained that they had wanted a much more direct approach to communications, which they also received:

Caregiver Model B: *we made it clear to them that we wanted […] to know fact* (context),* so [d]on’t try make us feel good* (response),* just tell us exactly what’s happening and what we can expect* (resource), *and we could work with that* (response). (FFC6)

RRS staff from Model B identified how they supported caregivers to improve their death literacy and reduce fear through managing expectations through skilled communication.

Rapid Staff Model B: *I think we expect a lot from families and carers* (resource) *[…] people are frightened by [death and dying]* (response)*, they’re worried by it, and they don't know what's normal* (context). (RS6)

The following caregiver explained how RRS staff undertook this communicative task of increasing knowledge and preparedness; helping him understand what was “*normal*”, i.e. improving their death literacy.

Caregiver Model B: *[RRS staff] kept us up to date at what was going on each stage of my mother's passing***,** *[…] until the end* (resource)*. “That's normal. Now, her throats closed” […], it's all a process that was explained to me* (resource) *[…]. They all knew the fears that my mother was in, [and] probably my naivety to death, ‘cause I didn't think death was like that, to be honest with you* (context). (FFC19)

All groups of participants agreed; having time with skilled communicators, who could share pertinent knowledge in a compassionate manner, could allay fears and so provide confidence and reassurance to patients and caregivers, this improved preparedness to care for caregivers. Ultimately, this can also avoid unnecessary hospital admission.

External HSCP Model A: *honest and frank communication* (resource)* [could] enable people to stay in their preferred place of care* (outcome)*, if that’s home, [through] providing answers* (resource)* to a lot of the fears* (context). (EX5)

Rapid Staff Model B: *[our communication] empowered the daughter to think ‘yes, I can do it [care for someone at home]'* (outcome). (SH6)

Whilst it was acknowledged that palliative and end-of-life care should be on all health care professionals’ agendas, our data suggested that it is specialist palliative care nurses and experienced health care assistants were best placed to undertake this form of skilled communication with patients and caregivers to ensure understanding during this challenging transition.

External Staff Model B: *[As] GP's, we do see [patients] at end-of-life [however], I would say the […] specialist nurses, […] they just get more of an understanding* (response) *[…]. I think that I've learned personally a lot from just going on visits with [palliative nurses]. […] So, I think it [is] everyone's sort of responsibility, but I think that the [specialist palliative care] nurses do it a lot better […] than anyone else I've seen* (resource). (EX8)

### Confidence, trust, and reassurance (mechanism- response)

Combined with the context, mechanism resources (i.e. time and skilled communication) elicit responses (i.e. particular types of reasoning), from those receiving the RRS. In this case these include both emotional and practical reactions of the patients and caregivers to the skilled communication of the RRS staff.

The following Model B RRS staff member explained how they were able to support a sense of control during their interactions with caregivers through the intentional building of relationships wherein effective and inclusive information sharing could take place.

Rapid Staff Model B*: [We] involve them in decision making *(outcome).* […] We explain about the anticipatory medications, 'cause sometimes they're given them, but they don't know what they are for* (resource).* They […] tend to say, “this is a deterioration, isn’t it? This is a sign?” And I'm saying, “it can be, however, […] [t]heir pain […] is controlled and it's about quality of care and quality of life and that's what we want* (resource).* And they agree with that* (response)*.* (RS7)

Where the RRS staff have the time and competency to provide knowledge in a compassionate and actionable manner, staff could improve caregiver preparedness and empowerment and help them to retain control.

Rapid Staff Model B*: [When caregivers] know what you're doing, why you're doing it* (response) *and they agree to it* (response), *[…] they feel involved* (outcome)*. And it's not like, they're not being able to care anymore, it's nothing to do with them, it's all the nurses. No, they are very much involved in everything that we do* (outcome). (RS7)

Participants discussed how skilled communication provided a sense of care and comfort throughout the end-of-life period and into bereavement.

Caregiver Model B: *[RRS] were just very respectful* (resource) *and if you did ask anything, they did explain it* (resource) *and they were very caring* (resource). (FFC18)

Rapid Staff Model B: [at VOED] *we’ve been there to [the] patient before, we know the family* (resource), *and then when we’ve finished the last offices we talk to the family […] and [ask] if you have any questions* (resource) *[…] people do tend to calm down* (response). (RS6)

Ultimately, skilled communication built reassurance and confidence to care for someone at the end-of-life, which led to a sense of preparedness in patients and caregivers, and so an improved experience of end-of-life at home.

Caregiver Model B: *they actually told us what to look for* (resource), *so we would [be] jumping in and picking up those signs in good time* (outcome) *[they] explained to us […] watch out for facial signs* (resource) *[…] it was nice to get that reassurance* (response) *[…] with compassion and care* (resource) *[…] the whole thing was made much easier* (outcome). (FFC6)

One caregiver from Model B explained they had been frightened to talk about death with the person who was dying. RRS staff helped them find the words they needed so they could have honest conversations.

Caregiver Model B: *[we first said to mum] you're looking good, you're on the mend, [RRS staff helped us to say], we're gonna care for you, we're gonna make sure you're comfortable* (resource),* [so] mum started coming round to who's gonna look after [dad], and then we realised that we were starting to say the right things* (response)* [...] And then that's when we would say we [will] look after him, we are here* (outcome) (FFC19)

In providing time and skilled communication (resources), RRSs encouraged reassurance and confidence in caregivers to provide care at home (responses), leading to subsequent outcomes of preparedness and empowerment and ultimately, death at home. It is these outcomes we outline in the next sub-section.

### Preparedness for end-of-life at home (outcome)

Where the RRS staff had elicited reassurance and confidence in both patient and caregiver, this led to several potential outcomes, including, a sense of preparedness, and/or empowerment to be able to ‘manage’ end-of-life at home.

Where access to the RRS had been difficult, or lost due to gatekeeping from the DN service, caregivers experienced a lack of this skilled communication delivered with compassion, which impacted on their knowledge and expectations, and so reduced their preparedness for death.

One caregiver in this position, had used the internet to try to find pertinent information.

Caregiver Model B: *My daughter was here. Because [wife] was really agitated through the day and couldn’t settle, and the daughter had learned somewhere* (resource) *that it could be a sign of not long left***.** (FFC11)

As a result, they had been left struggling to process what had occurred.

Caregiver Model B: *[S]he was talking, and she was normal in the morning. She felt sick all day, but […] I still wasn't expecting [death] *(outcome). (FFC11)

The rapid decline of this patient was unexpected to the caregivers and impacted on their ability to prepare and process the death. This experience highlights the necessity of the RRS being involved early enough in a patient's trajectory to be able to build relationships with the patient and caregiver, reducing the potential for gatekeeping barriers, and so delivering the tailored skilled communication available to those with access.

Another caregiver from Model B explained to us a delay in referral to RRS had meant that they were struggling to care at home using the DN service. When using the DN service only, they described having to wait long periods with their parent in pain as they struggled to get appropriate or timely responses to requests for help. Once they had access to RRS, this reduced the strain on her as a caregiver; without it, she explained: *we would have fallen over I think at the end of it* (outcome). (FFC6).

One RRS staff member walked us through the process they used to compassionately encourage preparedness.

Rapid Staff Model B: *It's extreme symptoms that cause most distress* (response),* […] this is why I tell families […] it's a bit like being born. Dying can be challenging. Dying can be difficult* (context). *[…] I always reiterate with families so that their expectation is [set]* (response)* […]. Yes, it will be, it can be, difficult, but, that we're just a phone call away* (resource) *and we'll come out* (resource) *and we'll try and help […] and explain things* (outcome)*.* (RS8)

The experiences of the last days of life can impact bereavement, skilled communication therefore mattered for those who are dying and those who are still living*.*

*External Staff Model A: We’re highly reliant on lay carers and family carers* (context),* and […] underestimate […] the need for them, and what [is] needed of them* (resource), *and what the result of that is [on] their bereavement* (outcome) *[…] positive or negative* (response). (EX4)

RRS staff had the experience, through their specialist role, alongside the opportunity through the service availability, to individually tailor communication; acting in a compassionate manner, reassuring and encouraging patients and caregivers, so they were not left in fear. Due to the time available to them, they could offer a prompt response, without the pressure of time limits on visits, to requests for help and support. Together, this encouraged preparedness in caregivers to manage the challenging period of end of life at home and better allow for enjoyment of the last days of life wherever possible during this final transition.

### Transitions theory

Aside from birth, our transition through dying and into death is perhaps the most notable. The impact of uncertainty, worry and complications, can have lasting impacts on those still living, including caregivers and indeed the health care professionals involved. In these circumstances, RRS staff make a positive impact to allay some of these concerns around death and dying at home.

Transitions theory allows us to recognise that a simple one-off event cannot support someone over a significant life transition. To undergo change/s and sustain wellbeing, support is required to prepare, manage and then move, into the next state. Transitional conditions provide us the space for thinking of wider contextual factors such as socio-economic conditions and personal and cultural differences. All can work to facilitate or provide barriers to healthy transitions [37, 38].

See supplementary file 5 for how we mapped the various aspects of transitions theory alongside the identification of contexts, mechanisms, and outcomes. A visual overview of the final refined programme theory, alongside transitions theory, is demonstrated in Fig. 1. The final refined programme theory is set out below in Table 3 and the aligned recommendations in Table 4.

**Table 3:** Final refined programme theory

**PT1 Skilled Communication**
Dying at home can be a difficult and challenging time for patients and informal caregivers, who may have had no or limited exposure to death and are fearful of it **(context).** When experienced nurses with compassionate communication skills can be flexible with time spent with families and caregivers **(resource)** this increases understanding and provides comfort and reassurance to patients and caregivers **(response),** who then feel a sense of preparedness and empowerment to manage dying at home **(outcome)**, which can improve the ability to appreciate this important and valuable final time together **(outcome)**

**Table 4:** Final recommendations

**Recommendations:**
• Practitioners should have a level of competence in effective communication which allows for shared understanding and care delivery
• Timely transition into palliative and end-of-life care is required in order to improve opportunities for patients and caregivers to receive tailored skilled communication, improving preparedness and addressing the fears surrounding death and dying
• RRS should be available and accessible to all who would benefit from inclusive, knowledgeable, and compassionate communication to support end-of-life at home

## Discussion

The challenges of low death literacy, and the associated fears and uncertainty around supporting death and dying at home, can be addressed, in part, by the availability and accessibility of a holistic and flexibly responsive RRS. Specialist staff in RRS’s have time available to them to attend to patients and caregivers at home as required, as a consequence of: not holding caseloads, being easily accessible through a direct telephone line, not having time limits on home visits, and their 24 hours 7 days a week response time. Where gatekeepers utilise, rather than block access to, the RRS, the specialist staff can employ their communication skills, imparting knowledge with compassion and including the caregivers, so they do not feel a loss of control. Skilled communication provides comfort, allays fears, and elicits reassurance and the confidence to care at home. Ultimately, this increases preparedness and improves short and long-term experiences for patients and caregivers at home at the end-of-life, while reducing use of other primary and community services.

Low death literacy is well established in the wider literature [9] alongside the work of clinical academics drawing popular attention to the lost understanding of the recognition and processes of ‘ordinary dying’ [13]. This lack of understanding sits alongside problematic media representations of death and dying (e.g., reporting of the Liverpool Care Pathway in the UK) compounding that fear. People are afraid due to their lack of knowledge and everyday experiences of death and dying and are afraid that those who care for them during death and dying may not have their best interests at heart [39]. Consequently, when patients and caregivers access the RRS at a challenging time, they are doing so with low levels of knowledge, are often unsure what to expect, and may well be fearful of ‘losing control’.

Our findings support another recent study with caregivers at the end-of-life [40]. The use of skilled communication in this study similarly supported caregivers to move towards 'accepting reality', encouraged the family into 'saying what you need to say', 'getting your house in order', and 'giving permission to die’ [40]. Knowledge of death and dying can embolden [41] and improve the confidence to care [42], reducing the ambiguity which can arise from a lack of practical and medical knowledge to both existential concerns and meaning making [18]. As others, we found compassionate and skilled communication led to death awareness and so death preparedness, for those who wished to receive it, which ultimately improved potential for dignity of the dying [43]. Further, that the extended potential for time and engagement with Model B allowed for increased access to the full dimensions of preparedness as set out in other research, including, medical, spiritual, psychosocial and practical concerns [44].

Compassionate communication demands high levels of emotional intelligence [45] and requires training and support to develop [46, 47]. For many caregivers, their interactions with the RRS team may be their first opportunity to discuss death and dying with a healthcare professional, since these difficult conversations are often missed [48, 49]. Our findings add that the skilled communication, provided by the RRS, was highly valued by patients and caregivers and improved their experiences while contributing towards a reduction in the use of other primary care services. Moreover, that the flexibility of the RRS means that those who would wish to be alone during the period of death and dying [7] can be. The RRS was individually responsive, those who required more support could have it, those who wished for less, the same, making it an effective and efficient service model.

Transitions theory [25], sets out the ways in which individuals cope with, understand, and manage change, as well as the way services can best support them through these. Where time is available to specialist staff, they can share valuable knowledge with compassion and kindness, supporting patients and caregivers through a significant transition, i.e. palliative to end-of-life care, active dying into death, death into bereavement [50]. All of which, if unsupported, has potential for harm. Timely transitions into palliative and end-of-life care matter for outcomes [51]. Our findings demonstrate that when there is early transition into community end-of-life care services, with specialist staff who have time available to care and communicate with compassion, the critical points of successful transitions can be safely addressed. These include a service needing to recognise and support through ‘different levels of awareness’, ‘critical points/milestones’, ‘loss of familiarity’, ‘questions about skills and capacities’, and ‘require new skills’ [25]. Our findings demonstrate that RRS staff have made a positive difference when they have supported caregivers to gain new knowledge, understand and recognise symptoms, and to change the way they are conversing with patients to allow for planning and goodbyes.

### What this research adds

Both Model A and Model B provided the opportunity for skilled communication, and so the ensuing positive benefits for patients and caregivers. Yet, we found overwhelmingly this was discussed more by Model B RRS staff and caregivers, perhaps due to the additional time available to RRS staff, including the support of a senior health care assistant (HCA) on home visits, and the more holistic approach of their service. As service commissioners consider the latest adult service specification for palliative and end-of-life care services from NHS England [52], it is worth bearing in mind our findings for the demonstrated value of time for skilled communications at home from specialist staff.

### Limitations and reflexivity

While our site gatekeepers worked industriously to obtain permission to contact details for us, it was difficult to then recruit these contacts into full participants (see Table 2). Informal feedback from several potential participants stated that when they received all the paperwork (invitation to interview, participant information sheet, consent form, health service use log, stamped addressed envelope) they found this overwhelming with too much in their personal life to focus on and consider. Although we assured them that the interviews and log did not all have to be completed, some preferred to withdraw at that point. As a research team, we made amendments to our ethics application, to make it clear in the information sheet that participants could participate in one interview only, and/or complete a log. We acknowledge that we were looking to interview caregivers who were at a vulnerable time in life and recognise that this is an issue seen across end-of-life and bereavement research [53, 54].

We recognise that the issues with recruitment led to a relatively small sample, and the demographics of our sample somewhat matches the criticism in the wider literature [55, 56] that end-of-life care services are most often accessed by white, British, middle class service users. We would wish to address this in future research.

### Further research

A fundamental aspect of the communications skills and the benefit these provide discussed in our paper are based on an evaluation which predominantly engaged with participants with knowledge of, or who had engaged with, the services. Given what we have learned from our study on the impact and value of this communication to those who receive it, we suggest further research is needed into the cultural competencies required of the services to engage all communities who would wish to benefit, and how this would recognise and respond to differences in different populations, ethnicities and cultures and their goals for end-of-life care [57–60].

## Conclusions

Our findings demonstrate the availability of experienced specialist palliative and end of life care staff to patients and caregivers at home at the end-of-life, in a flexible manner to suit individual preferences, makes a tangible and positive difference to short- and long-term outcomes. The identification of end-of-life, active dying and death, and support into bereavement, required appropriate and timely referrals into RRS. Once referred in, RRS staff were able to rapidly build relationships and provide compassionate support to patients and caregivers, though the often uncertain and extraordinary experience of end-of-life care at home. The flexibility of the RRS, provided for an individually tailored response, so those who wanted more contact could receive that, and those who wanted less contact, the same. The lack of time restrictions on home visits and the rapid response to requests for help provided the space for the provision of skilled communication which presented as ‘professionalism with compassion’.

## Supplementary Information

Supplementary Material 1: Supplementary File 1. Realist Evaluation Reporting Tool.

Supplementary Material 2: Supplementary File 2. Overview of Development of Initial Programme Theory (IPT), through identification of various contexts, mechanisms and outcomes (CMO) through to final prgramme theory (PT) for PT1 Skilled Communication.

Supplementary Material 3: Supplementary File 3. Interview Topic Guide/Schedule for RRS/External.

Supplementary Material 4: Supplementary File 4. Interview Topic Guide/Schedule for Caregiver.

Supplementary Material 5: Supplementary File 5. Copy of Matrix and Mapping for Elements of Transitions Theory (TT) against IPT areas.

## Data Availability

The datasets used and/or analysed during the current study are available from the corresponding author on reasonable request.

## Abbreviations

COPD: Chronic Obstructive Pulmonary Disease
CMO: Context Mechanism Outcome
DNR: Do Not Resuscitate
DN: District Nurse
GP: General Practitioner
IPT: Initial Programme Theory
PT: Programme Theory
PPI: Public Patient Initiative
RRS: Rapid Response Service
VOED: Verification of Expected Death
