# Supplementary material for: Providing ‘professionalism with compassion’; how the time for caring communication can improve experiences at the end-of-life at home, findings from a realist evaluation
Source: BMC Palliat Care. 2024 Dec 21;23:287. doi: 10.1186/s12904-024-01610-4 (PMC11662561; doi:10.1186/s12904-024-01610-4)
Supplement: Supplementary file 2 — Supplementary Material 2: Supplementary File 2. Overview of Development of Initial Programme Theory (IPT), through identification of various contexts, mechanisms and outcomes (CMO) through to final prgramme theory (PT) for PT1 Skilled Communication. [file 12904_2024_1610_MOESM2_ESM.docx]

**Supplementary File 1 – IPT-PT Development**

This supplementary file outlines the development of the final refined programme theory in the area of Communications. This begins at the initial programme theory (IPT), through stages of analysis to investigate several potential differing context, mechanism, and outcomes (CMOs), to a revised programme theory, and then a final programme theory (PT)

The IPT in this area stated:

**IPT Communication** If patients and their families have the opportunity (context) to receive open and timely communication about the RRS at the end of life (what it is, what it offers, when and why) (resource), they can make an informed decision about death and dying at home (reasoning) and so will self-refer to the RRS (outcome-1), and/or post-referral (self or DN/GP) use the RRS, as opposed to another emergency service, in times of need (outcome-2), and admissions to hospital at end of life will be reduced (outcome-3).

**Key point:**

Open and timely communications, that are clear and appropriate, support people to engage with the RRS and provide valuable information as service users move through transition(s) into end-of-life care and through end-of-life care period into death/post-death period.

**Definition of ‘communications’:**

It became clear that the concept of communications could be conceptualised several ways; i) rapid response services internal communication between the service and service staff, ii) rapid response service external communications for example to DNs, GPs, Emergency Services, Discharge Teams, Hospices, and the General Public, including patient information sharing or service promotion, or iii) rapid response staff communications to patients and caregivers, either by telephone or on home visits, that shares knowledge and information about how to act, care, cope, and otherwise manage the processes of death and dying at home. We agreed through early information gathering that we would focus on the third conceptualisation; that being, the active process of communication between rapid response staff and patients and caregivers and the impact of that communication. This decision was made in part as the investigation of referral was best addressed under the other explanatory area of ‘Service Access’. To demonstrate the specific form of communication we are discussing, we extended the title of the PT from ‘Communication’ to ‘Skilled Communication.’

**Additional CMOs Developed Analysis Round 1:**

As theory development continued, the original IPT was tested against the data through iterative stages of analysis. Several context, mechanism, outcome configurations (CMO) were identified as emergent. All CMO for this analysis round for this IPT area are set out below.

**CMO COMMUNICATION: HONESTY**

**CONTEXT/S:** service users are **unaware** of gravity or reality of the situation- i.e., that the patient is at end of life, AND/OR service users are **unprepared** for awareness of the gravity or reality of the situation- i.e., that the patient is at end of life

**MECHANISM/S:** the staff use their time/flexible verbal/non-verbal communication skills to provide knowledge and psycho-social support that is appropriate to that service user (i.e., may or may not use 'death'- and cannot give an 'expiration date' as RS4 explained) (costs: expert skills/knowledge/training)

**OUTCOME/S:** ***positive***- service users better understand the situation and can prepare and adapt, their expectations are better managed, and consequently they are reassured and better 'prepared' to care (benefits: patients feel reassured/empowered, positive impact on well-being). ***negative***- this cannot always be managed (as RS5 syringe driver) although will try best to manage.

**CMO COMMUNICATIONS: CHAOTIC AND TURBULENT COMMUNICATIONS**

**CONTEXT/S:** managing expectations and/or understanding different needs and concerns of a full household is difficult at the end of life - particularly where situations, and so 'preparedness', may change over time (costs: staff having time prior to visits e.g., read information/preparation, multiple visits/contact)

**MECHANISM/S:** staff use their time/flexible verbal/non-verbal communication skills to support difficult household communications, managing tension and competing needs and desires, supporting all to be heard and understood (costs: staff training/time; benefit: families being heard/part of decision-making process)

**OUTCOME/S:** service users better understand the situation and can prepare and adapt, their expectations are better managed, and consequently they are reassured and better 'prepared' for whatever comes next - be that end of life at home or in hospice (costs: reduction in unplanned care e.g., emergency admissions, ooh GPs etc; benefits: increased reassurance/interactions/experience/empowerment)

**CMO COMMUNICATIONS ‘EXPERT COMMUNICATIONS’**

**CONTEXT:** Because of low death literacy and the intensity of the emotional and physical experience of supporting someone to die at home … (costs: staff having time prior to visits e.g., read information/preparation, multiple visits/contact, increased costs for more qualified ‘expert’ staff (i.e., higher salary/training costs for grade 7) and/or additional HCA staff)

**MECHANISM/S:** repeated expert communications are required on an 'as and when required' basis, considering the 'triad' of experts involved which leads to appropriate and timely sharing of information that helps manage expectations (costs: training, multiple visits/travel and multiple staff)

**OUTCOME/S:** this leads to positive outcomes for service users/service (costs: reduction in unplanned care e.g., emergency admissions, ooh GPs etc; benefits: increased reassurance/interactions/experience/empowerment)

**CMO COMMUNICATIONS ‘STAFF AVAILABILITY/MODELS/TIME’**

**CONTEXT/s:** complex (chaotic, concerned, distressed households).

**MECHANISM/s:** two members of staff in DRRS- can manage multiple aspects of care and multiple household members at once - so can provide pain relief + communicate for reassurance to FFC simultaneously (costs: additional cost of two staff members rather than 1 e.g., NRRS)

**OUTCOME/s:** managing multiple aspects of care with positive outcomes for patient and FFC (Costs: for NRRS – cost of providing multiple aspects of care and outside 8-7pm, what cost and to whom? Benefit: less calls to/input from GP/DN; Benefits: more streamlined/specialist care, reduction in the use of emergency/ooh services and hospital admissions)

**CONTEXT/S:** where there is time available for the RRS staff to ascertain need and appropriateness (costs: staff having time prior to visits e.g., read information/preparation, multiple visits/contact)

**MECHANISM/S:** staff can 'teach' or provide knowledge and care activity to FFC at home (benefits: increased reassurance/empowerment)

**OUTCOME/S:** positive outcomes for patient (e.g., comfort from mouthcare) and for FFC (e.g. inclusion and understanding leading to improved well-being in short and longer term) (costs: increased time spent with patients/caregivers; benefit: families being reassured and confident to provide care/positive interactions with RRS)

**CONTEXT/S:** where there is time available for the RRS staff to ascertain need and appropriateness (costs: staff having time prior to visits e.g., read information/preparation, multiple visits/contact; staff training/awareness)

**MECHANISM/S:** staff can build a relationship with patient and FFC to understand differing needs and so provide a culturally sensitive package of care (benefits: increased reassurance/empowerment)

**OUTCOME/S:** respect, engagement, positive outcomes from completing appropriate rituals (costs: increased time spent with patients/caregivers, training/awareness; benefit: families being heard/reassured/understood and positive interactions with RRS)

**PT Development Analysis Round 2:**

Through the second round of analysis these CMOs were further interrogated against the data and were drawn together into one overall group for consideration and revision.

**RESOURCE or CONTEXT:** SKILL (excellent comm skills+specialist knowledge) **RESOURCE or** **CONTEXT:** TIME (early referral/in home visits and on phone/repeated visits) **RESPONSE/OUTCOME**: ALLOWS FOR HONEST AND CLEAR COMMUNICATIONS - WHICH SUPPORTS CAREGIVER PROVIDING PREPAREDNESS AND UNDERSTANDING AND ABILITY TO RECEIVE/PROVIDE CARE – AND ULTIMATELY SUPPORTS ABILITY TO DIE AT HOME.

Specialist nurses, supported by a senior HCA where possible, have excellent communication skills and extensive knowledge (CONTEXT), where they are able to spent time with patients and caregivers (early referral into the service/time available in a home visit/repeated visits to build rapport) (RESOURCE), they can provide expert advice, support and guidance to caregivers (RESOURCE), which leads to preparedness, understanding, and an improved ability to receive/provide care (RESPONSE), this supports the ability of the patient to die at home (OUTCOME-1) and improves well-being of caregiver and their transition into bereavement (OUTCOME-2).

**Final Revised PT Following Integrations of Substantive Theory and Research Team Agreement:**

Over the course of the analysis and early attempts to revise the programme theory, we discussed the use of several different adjectives before the identification of communication skills as a resource. These included: ‘advanced communication skills’, which was rejected as it might imply a particular course or qualification which had been undertaken by staff; also, ‘specialist communication skills’, which we agreed implied the same; in the final analysis therefore, ‘expert communication skills’ was agreed for the final programme theory. This aligns well with wider literature, e.g., ‘intelligent kindness’ (Campling, 2015) and ‘compassion’ (Kellehear, 2007; 2014).

These communication skills are intentionally identified as a professional competency, rather than solely as a personal attribute of the rapid response service staff member. This competency means that those who possess expertise in end-of-life care can share pertinent knowledge and information with patients and caregivers in an appropriate, person-centred way that makes a positive difference to them. Differences can be made through reduction in burden of death and dying, through the reduction in the burden of treatment (May, 2014). This helps the transitions (Melsis, 2014) throughout and overall, rather than as an outcome.

**PT1 Skilled Communication**

Dying at home can be a difficult and challenging time for patients and informal caregivers, who may have had no or limited exposure to death and are fearful of it **(context).** When experienced nurses with compassionate communication skills can be flexible with time spent with families and caregivers **(resource)** this increases understanding and provides comfort and reassurance to patients and caregivers **(response),** who then feel a sense of preparedness and empowerment to manage dying at home **(outcome)**, which can improve the ability to appreciate this important and valuable final time together **(outcome)**.

**Recommendations:**

- Practitioners should have a level of competence in effective communication which allows for shared understanding and care delivery.
- Timely transition into palliative and end-of-life care is required in order to improve opportunities for patients and caregivers to receive tailored skilled communication, improving preparedness and addressing the fears surrounding death and dying.
- RRS should be available and accessible to all who would benefit from inclusive, knowledgeable, and compassionate communication to support end-of-life at home.
