# Supplementary material for: Providing ‘professionalism with compassion’; how the time for caring communication can improve experiences at the end-of-life at home, findings from a realist evaluation
Source: BMC Palliat Care. 2024 Dec 21;23:287. doi: 10.1186/s12904-024-01610-4 (PMC11662561; doi:10.1186/s12904-024-01610-4)
Supplement: Supplementary file 3 — Supplementary Material 3: Supplementary File 3. Interview Topic Guide/Schedule for RRS/External. [file 12904_2024_1610_MOESM3_ESM.docx]

##
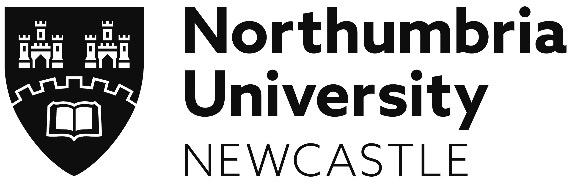


| **Interview Topic Guide– For HSCPs working in or with the Rapid Response Services**  Rapid Response Service Models in End of Life Care |
| --- |

**Please commence recording as soon as you begin the interview – including the preliminary discussion, checking first that the participant is happy with this.**

**Step 1: Introduction**

1. Self-introduction

“Good Morning/Afternoon/Evening. Thank you for agreeing to be interviewed. My name is…… I am an academic at University of Northumbria at Newcastle and one of the research associates on the study.”

2. Consent & Confidentiality

“Please could I check that you have read and understand the participant information sheet and the consent form?

The purpose of this study is to investigate how Rapid Response Services in end-of-life care work, for who, and why. The information you provide today will be used to produce publications such as reports, papers, and presentations to help with the future planning of these services. Rest assured that everything you say is confidential and you will not be named in any report/paper to come out of the study.

Only the research team will have access to this recording, which will be deleted once transcribed. All data will be anonymised as soon as possible following collection, and all identifiable details destroyed once analysis is complete. All data will be kept securely on University of Northumbria at Newcastle research repository for 7 years after the research is complete. No data will ever be held on researchers own devices. If this is recorded on an external recording device, the file will be moved to the secure server and deleted off the device as soon as possible after the interview has taken place.

It’s likely that the interview will take between 60-90 minutes (but can be longer or shorter than this; I will be led by you), and it will include questions about your understanding and experiences of the Rapid Response Service. Please let me know if you’d like to stop at any time for a break or if you would prefer to stop the interview completely. And to reiterate, you are free to withdraw from the study up to 28 days after this interview and you have the contact details to request that on your participant information sheet.

This study has been granted ethical approval by University of Northumbria at Newcastle Ethics Committee and the Health Research Authority. If you have any complaints or concerns, you have full contact details for me and the wider team in the participant information sheet.

Before I start, just to make clear, that we really want to listen and hear your views about the Rapid Response Service and that what you say will be confidential.

**“Can I confirm that you consent to take part in the interview and for the interview to be recorded?”**

**“Have you any questions before we start?”**

**Step 2: Questions**

“Please feel free to talk about anything related to my questions: there are no right or wrong answers……”*.*

I’d like to start by asking you some questions to help us to understand your current situation (first, ask participant how they’d like to be referred to).

**Role**

Please could you tell me something about your current role working for/with the Rapid Response Service?

*Prompts:*

*What is your title?*

*How long have you been working in your role?*

*What are the best and most challenging parts of your role and responsibilities?*

*How do you feel about the service you provide? To patients? To carers?*

Over the remaining part of the interview, I will present 8 different topic areas to you to guide our discussion. For each topic, I will share in one or two sentences what the research team think *could* be happening and what *might* be important in one aspect of the Rapid Response Service. We will then ask you to share your expert reflections on our statement, i.e., does it feel right? What is missing? How would you explain it? including your own examples wherever possible.

**COMMUNICATIONS**

**When patients and caregivers receive open and timely communication about the RRS they are more likely to use the service**

Is this your experience? Can you share any examples?

**PROMPTS**:

1. Covered what RRS does, how, when, and why?

2. Covered what to expect (into end of life- in active dying- post bereavement)?

3. Encourages self-referral or engagement post referral?

**Original Questions:**

1. What do service users receive? When? How do they respond?
2. Are communications tailored to different individuals/families (language/form/information)?
3. Are they fully informed and prepared as a result?
4. What works well? What doesn’t work so well? What is missing?

**ACCESS**

**Referrals into the RRS can sometimes be difficult due to barriers such as people, services, and systems, this may lead to a lack of use of RRS and increased hospital admissions.**

**Is this your experience? Can you share any examples?**

**PROMPTS:**

1. Referral routes: GP, DN, Palliative Teams, Discharge Teams?
2. People/Services/Systems that help/hinder?

**Original Questions:**

1. What is a clear, transparent, and unconstrained pathway? What are barriers to this?
2. How do referral pathways include? How do referral pathways exclude?
3. What other services are referred to/used concurrently? (i.e., sitting services?)

**VALUES**

**Community end of life care services tend to be used more often by white, middle class, service users. Some people may seek care from elsewhere (family, faith group, community) as their beliefs or background don't 'align' with the Rapid Response Service.**

Is this your experience? Can you share any examples?

**PROMPTS:**

1. Do you think the RRS is accessed and 'open to' those from different socio-economic/social class/LGBTQ+/religion/ethnic backgrounds?

2. What could be done differently to include more people from all backgrounds in community end of life care services?

**Original Questions:**

1. What are the hidden values of the RRS? What are the open expectations of the RRS? (socio-economic/LGBTQ+/social class/religion/ethnicity)
2. How might patients/caregivers feel included by these? How might patients/caregivers feel excluded by these?

**DIVERSE NEEDS**

**Historically, community services to support end of life care at home were developed to support those with a cancer diagnosis. Consequently, those with multi-morbidities, non-malignant conditions, frailty, and dementia, may not access and engage with the RRS, and so will be more likely to be admitted to hospital.**

Is this your experience? Can you share any examples?

**PROMPTS:**

1. How/Does age, pain, multi-morbidities, frailty, dementia impact on service use?

**Original Questions:**

1. Do you agree or disagree with this statement? Can you share any examples?
2. What needs does the RRS meet for patients/caregivers? Should they meet more or fewer than this? Are there gaps in service?
3. Why are community end of life services most often accessed by those with malignant disease?
4. How are pain/multi-morbidity/demented/frailty barriers to community end of life care?

**GEOGRAPHY**

**Where people live, in the countryside or in the city, can impact on their access and experience of the RRS. In times of crisis, patients and caregivers location in relation to the RRS and other services influences their choice to use the RRS**

Is this your experience? Can you share any examples?

**PROMPTS:**

1. Does it make a difference to i. service access ii. choice to use service iii. referral into service if someone is in an urban or rural location?

2. Collaborations/partnerships? ‘Just in Case’ boxes?

**Original Questions:**

Same as prompts

**10v24hr CARE**

**24hr: The knowledge that a service can be accessed during ‘the ordeal of the night’ reassures patient/caregiver during the day and reduces emergency admissions.
10hr: By planning day services ‘the ordeal of the night’ can be avoided through preparation reducing emergency admissions without requiring a 24hr service.**

Is this your experience? Can you share any examples?

**PROMPTS:**

1. Benefits/Challenges of 24hr
2. Benefits/Challenges of 10hr

**Original Questions:**

Same as prompts

**TIMELINESS**

**Where the RRS guarantees a timely (within one hour) and appropriate (attendance if required) response, patients and caregivers feel reassured and will choose to use the RRS, reducing use of emergency services and hospital admissions.**

Is this your experience? Can you share any examples?

**PROMPTS:**

1. What is timely?
2. What are the different responses? (phone/video/email/visit)
3. What response is appropriate from the RRS and for what? (i.e. symptoms, psychological, etc)?
4. Why do you think carers contact the RRS?

**Original Questions:**

1. What is the optimum response time for: answering service requests – does this change for differing forms of service requests?
2. What is the optimum ‘response’ for differing service requests? (phone/video-call/email/home visit)?

**WHO**

**Who is at home (caregiver + other family/dependants) and what skills/needs they have, influences engagement with the RRS. Similarly, who is coming in from the service (staff members competencies/capabilities/cultural awareness) influences service user engagement with the RRS.
There needs to be a match between who is at home and who is coming into the home for optimum outcomes**

Is this your experience? Can you share any examples?

**PROMPTS:**

1. What skills/capabilities/responsibilities are required of the caregivers at home?

2. Does it impact on service use if there are other dependents in the home? How so?

3. What skills/capabilities/responsibilities are required of the RRS staff attending the home?

4. Does there need to be a ‘match’ between those at home and those coming into the home?

**Original Questions:**

Same as prompts

**Step 3: Closing**

**“I’ve nearly finished the interview.** **Is there anything you’d like to add before I finish?** Thank you very much for your contribution. Please feel free to contact the team if there is any further information you need about the study….”

Offer to send a study summary once we’ve finished…….

**Would you like to receive a summary of the outcomes of the study?**

**During the Interview** - if people become upset or appear tired, allow them time to think, pause, cry, rest – ask them if they want to carry on or take a break or reschedule or stop completely.
