# Supplementary material for: Providing ‘professionalism with compassion’; how the time for caring communication can improve experiences at the end-of-life at home, findings from a realist evaluation
Source: BMC Palliat Care. 2024 Dec 21;23:287. doi: 10.1186/s12904-024-01610-4 (PMC11662561; doi:10.1186/s12904-024-01610-4)
Supplement: Supplementary file 4 — Supplementary Material 4: Supplementary File 4. Interview Topic Guide/Schedule for Caregiver. [file 12904_2024_1610_MOESM4_ESM.docx]

##
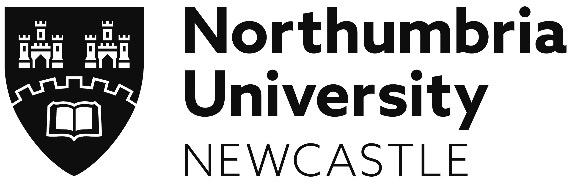


| **Interview Topic Guide – [For carers whilst using the Rapid Response Services]**  Rapid Response Service Models in End of Life Care |
| --- |

**Please commence recording as soon as you begin the interview – including the preliminary discussion.**

**Step 1: Introduction**

1. Self-introduction:

“Good Morning/Afternoon/Evening. Thank you for agreeing to be interviewed. My name is…… I am an academic at University of Northumbria at Newcastle and one of the researchers on the study.”

2. Consent & Confidentiality:

“Please could I check that you have read and understand the participant information sheet and the consent form?

The purpose of this study is to investigate how Rapid Response Services in end-of-life care work, for who, and why. The information you provide today will be used to produce publications such as reports, papers, and presentations to help with the future planning of these services. Rest assured that everything you say is confidential and you will not be named in any report/paper to come out of the study.

Only the research team will have access to this recording, which will be deleted once transcribed. All data will be anonymised as soon as possible following collection, and all identifiable details destroyed once analysis is complete. All data will be kept securely on University of Northumbria at Newcastle research repository for 7 years after the research is complete. No data will ever be held on researchers own devices. If this is recorded on an external recording device, the file will be moved to the secure server and deleted off the device as soon as possible after the interview has taken place.

It’s likely that the interview will take between 30-40 minutes (but can be longer or shorter than this; I will be led by you), and it will include questions about your understanding and experiences of the Rapid Response Service. Please let me know if you’d like to stop at any time for a break or if you would prefer to stop the interview completely. And to reiterate, you are free to withdraw from the study up to 28 days after this interview and you have the contact details to request that on your participant information sheet. This is the first of two interviews, we will speak again in the future and wherever possible we will ensure the interviews are with the same research associate.

This study has been granted ethical approval by University of Northumbria at Newcastle Ethics Committee. If you have any complaints or concerns, you have full contact details for me and the wider team in the participant information sheet.

Before I start, just to make clear, that we really want to listen and hear your views about the Rapid Response Service and that what you say will not affect the service you, or the person you care for, receive.

**“Can I confirm that you consent to take part in the interview and for the interview to be recorded?”**

**“Have you any questions before we start?”**

**Step 2: Areas for questions**

“Please feel free to talk about anything related to my questions: there are no right or wrong answers……”*.*

**We would like to start by asking some ‘about you’ questions.**

***About you***

1. What is your post-code?
2. What is your occupation [was if retired]?

1. What is your gender identity?

Male

Female

Other

Prefer Not To Say

***Service Rating***

1. Please could you tell me if the services you have received have:

Exceeded your expectations

Just met your expectations

Fell short of your expectations

***Personal Circumstances***

1. How they would like to be referred to (e.g., carer/caregiver/by familial association/etc.)

***IPT Area Questions***

| **IPT1 Communications**  If patients and their families have the opportunity (context) to receive open and timely communication about the RRS at the end of life (what it is, what it offers, when and why) (resource), they can make an informed decision about death and dying at home (reasoning) and so will self-refer to the RRS (outcome-1), and/or post-referral (self or DN/GP) use the RRS, as opposed to another emergency service, in times of need (outcome-2), and admissions to hospital at end of life will be reduced (outcome-3). |
| --- |

1. Sometimes, **RRS staff will discuss with caregivers what to expect when supporting someone at the end of life at home, and having that information makes them feel better prepared** to manage different symptoms and changes. Is that your experience?
2. There can be lots of services involved when you’re supporting someone at the end of life at home. **Did the RRS staff help you know when to contact them and when to contact other services like your GP or your DN**?
3. Sometimes care givers feel like they can’t ask all the questions they have when they speak to RRS staff. Maybe due to time or as it doesn’t feel right to ask. **Do you always feel you can ask everything you might want as many times as you need to?** (*prompt: examples?)*

| **IPT2 Values**  Rapid Response Services display implicit and explicit values and expectations to potential service users (context-resources), where there is some misalignment of values and expectations for patients/carers, they feel fear/dislike/distrust and/or that they will be poorly judged by the service (mechanism-reasoning), and this leads them to choose not to use the service and/or seek assistance elsewhere, which could result in more hospital admissions (outcome) |
| --- |

1. From the discussions we have had so far, we have learnt that supporting people at the end of life at home can mean getting help with beds, bedding, laundry, heating costs, time off work, or time off caring to rest. **Can you tell me what you have needed help with? (Is there anything more that would help you?**)
2. Sometimes, patients and their caregivers can be afraid of **losing control or losing privacy at home when using end of life care services**. How do you feel about RRS staff coming into your home and helping you?
3. IF NEEDED FOR MORE INFO: We know that it can be a worry inviting someone into your home at such a difficult time, and when someone is so unwell. **Did you ever have any concerns about having the RRS in your home**?
4. Our different faiths, religions and cultures can mean we want to do things at home in particular ways. **How did the RRS staff take all your needs into account?** (*prompt: would/did it influence your decision to use?)*

| **IPT3 Access**  When pathways into the Rapid Response Service (RRS) are clear, transparent, and unconstrained to all that could benefit from the service (context), patients are able to access the immediate care that RRS offers if necessary (resource) and therefore feel confident to provide care at home (reasoning) which leads to less emergency calls (outcome 1) and more home deaths (outcome 2). |
| --- |

1. Sometimes people first find out about the RRS from a leaflet they’ve picked up, or maybe their GP or DN has helped them access the service. **How did you first access the service?** (*prompt: any pros/cons of that?*)
2. Sometimes, patients and carers feel like they want RRS staff to be their key worker, meaning the RRS becomes their first point of contact when they need support. **Sometimes people prefer that key worker to be their GP, another specialist nurse, or district nurse that they have already got to know well.** What was your experience?
3. Some people have told us that having RRS support helped them to care at home. **So having a telephone number to ring, where someone can come out straight away makes a difference**. Is that your experience?
4. Do you think you would have been able to care at home**, had you not had the support of the RRS, and maybe just had standard prearranged appointments**?
5. Our research suggests that when people don’t have support from something like the RRS they can often panic and call for emergency services. **Was there ever a time when you felt unsupported or felt the need to call for emergency services?**

| **IPT4 Diverse Needs** Historically, community palliative care services were developed to support patients with cancer to die at home (context), now guided by the GSF and ACP (resource), professionals can demonstrate understanding of, and sensitivity to, the diverse needs of individuals (reasoning), and so appropriate referrals are made for those approaching end of life, regardless of diagnosis (outcome), but societal norms can stop people from engaging (context) |
| --- |

1. What illness does the person you care for have that has led to them using the RRS?
2. **Do you think there is anything about this illness, its symptoms, or the care it requires that has made it more difficult to use the RRS?** (*prompt: how so? Follow up any responses to ask for examples)*

| **IPT5 Geography + Community**  Typically, older population profiles mean that living and dying in rural areas is by no means a homogenous experience (Hospice UK, 2021). The geographical spread of patients in rural areas is vast, meaning that a one-hour response is not always feasible (context), therefore when there is no timely response from RRS (within one hour) (resource), and family carers feel unsupported and unconfident to provide end of life care (EoLC) to their family member (reasoning), this leads to a higher likelihood of emergency phone calls and admissions (outcome). |
| --- |

1. Sometimes where people live can impact on their choice to use the RRS. **They might feel it takes too long to get to them, or they are in a community which takes care of each other themselves**. Is that your experience?

| **IPT6 24v10hr** Previous research (Dalkin et al, 2018) indicates that family members feel less supported and more vulnerable at night (context), where the RRS provides 24 hours care (as opposed to 10 hour care model) (resource), family carers feel confident to provide end of life care (EoLC) to their family member at home in the knowledge that they can access care overnight (reasoning), this leads to a reduced likelihood of emergency phone calls and admissions from a 24 hour service, as opposed to a 10 hour service, and so the patient preference for a home death is met (outcome).  **Rival:** 10hr services are aware of the limitations of their provision (context) and therefore work proactively to ensure planned care that equips carers with strategies (resources) to make sure they can cope overnight (reasoning) resulting in less emergency admissions and more home deaths (outcome). |
| --- |

1. Some services are only available 10 hours, 9-7pm, whilst others are 24 hours a day. Your service was (10hr/24h):

**10hr**

**Do you think it would have been beneficial if this service had been 24 hours** instead and how would that have helped you?

**Were there any instances when you wanted to phone RRS but they weren’t open**?

**24hr**

Did you call the RRS out of hours? **Did it ease your anxiety knowing that the service was available if you needed it?**

| **IPT7 Timeliness** Death has become hidden from society and medicalised, but home deaths are the preference of many individuals (context), where there is a timely response guaranteed from RRS (within one hour) (resource), patients and carers feel reassured, supported, and confident to provide end of life care (EoLC) at home (reasoning), and this leads to reduced numbers of calls to emergency services and less emergency admissions (outcome).  **Rival**: Carers perceive the service to be a rapid response line (context) where these expectations are not met (resource) carers lose trust in the service and feel let down (response) which leads to increased carer burden (outcome) and/or increase in emergency calls (outcome). |
| --- |

1. The **RRS attempts to answer or return calls with a one-hour response time**, is that your experience? (*prompt: what difference did that make? Examples?)*
2. The RRS can respond to a call for help by answering queries on the phone and they can also make home visits**. When you’ve contacted them for help were you satisfied you got the right response (phone call/home visit)?**

| **IPT8 Who (at home/from service)** The provision of EoLC at home requires internal and external support (context), when the internal supporter(s) (FFC) is confident and committed to EoLC at home (as perceived by themselves and the patient) (resource-reasoning) and trusts in the skills, supplies, service, personhood, of the external support (RRS) (resource-reasoning), the RRS will become the service of choice, avoiding use of the emergency services or DN's (outcome-1) and/or supporting death at home (outcome-2) |
| --- |

**HOME:**

1. From what we have learned so far, we understand there are lots of practical arrangements people need to manage when caring for someone at the end of life at home. Could I ask **what, if anything, you have had to change or adapt in your life or home and what that has meant for you?** (*prompt: living arrangements, work, care)*

**STAFF:**

1. RRS staff are specialists in end of life care, they should have enhanced knowledge and understanding of end of life and more time to spend on home visits. We understand that you have lots of interaction with lots of people when caring for someone at home though. **Did you notice the differences between these specialists and other HSCP?**
2. Sometimes**, patients and caregivers feel like they have been able to build a relationship with the RRS staff,** is that your experience? (*prompt- can you give any examples of the (dis-)benefits of (not) having that?)*
3. *Different question per area:*

**NRRS:**

**In your area, RRS staff do home visits as a single member of staff,** sometimes that’s preferable as its less people in your home and you can get to the know them, sometimes people prefer staff to come in two’s so there is more helping hands. How do you feel about that? *(prompts: examples)*

**DRRS:**

**In your area, RRS staff do home visits in a team of two,** that means they have more time and hands available for care and support, sometimes people prefer when staff come as single workers as then it feels quieter and easier to talk and build a relationship. How do you feel about that?

**Step 3: Closing**

**“I’ve nearly finished the interview.** **Is there anything you’d like to add before I finish?** Thank you very much for your contribution. Please feel free to contact the team if there is any further information you need about the study….”

If pre-bereavement:

**This was the first interview of two I will be in contact to speak to you again in the future although you do not have to continue if you don’t prefer not to.**

Offer to send a study summary once we’ve finished…….

**Would you like to receive a summary of the outcomes of the study?**

Once the interview has completed, please state the day, date, and time of interview – so it is recorded – before turning off the recording.

**During the Interview**

If people become upset, allow them time to think, pause, cry – ask them if they want to carry on or take a break or reschedule or stop completely. Ask if they have someone else to talk to. Suggest the contact numbers that have been shared with them by RRS if they need further support.
